# Follow-Up for Subjective Memory Concerns Expressed During the Medicare Annual Wellness Visit

**DOI:** 10.1093/geroni/igaf122.1067

**Published:** 2025-12-31

**Authors:** Aleksandra Wec, Mingche Wu, Danny Scerpella, Zhang Zhang, Danielle Peereboom, Jennifer Wolff, Danielle Powell

**Affiliations:** Johns Hopkins Bloomberg School of Public Health, Baltimore, Maryland, United States; Johns Hopkins University, Baltimore, Maryland, United States; Johns Hopkins Bloomberg School of Public Health, Baltimore, Maryland, United States; Johns Hopkins Bloomberg School of Public Health, Baltimore, Maryland, United States; Johns Hopkins Bloomberg School of Public Health, Baltimore, Maryland, United States; Johns Hopkins University - School of Public Health, Baltimore, Maryland, United States; University of Maryland, University of Maryland College Park, Maryland, United States

## Abstract

The Medicare Annual Wellness Visit (AWV) includes a health risk assessment (HRA) with questions about subjective cognitive function and memory. Our objective was to identify the frequency of follow-up actions in response to newly reported memory concerns during an AWV. Using electronic health record (EHR) data from an academic health system spanning October 2017–October 2022, we identified AWVs where patients first reported memory concerns in the HRA, excluding cases with prior diagnoses of dementia or mild cognitive impairment. Using visit notes, Current Procedural Terminology (CPT) codes, and ICD-10 codes from relevant encounters, we assessed frequency of relevant follow-up actions, including memory screenings, referrals for specialist care (memory care, neurology, geriatric psychiatry), dementia diagnosis, and advanced care planning (ACP).  Our sample included 1,801 AWVs for 1,411 unique patients. Patients were predominantly white (70%) and female (63%), with an average age of 78 (SD 7.48). Memory screeners (e.g., Mini-Mental Status Exam) were documented in the visit notes of 4.7% AWVs and 1.3% of AWVs resulted in a referral to a specialist. In total, 4.5% of patients had a new dementia diagnosis documented in their record during the AWV and 10.6% received a newly documented diagnosis in the 12 months following the visit. ACP was completed concurrently with 28% of AWVs. Our study demonstrates the feasibility of using the EHR to identify actions following emerging memory concerns expressed during the AWV. Health systems may use the findings to identify targets for improvement in dementia care as part of the AWV.

